# Adaptive downregulation of Cl^-^/HCO_3_^-^ exchange activity in rat hepatocytes under experimental obstructive cholestasis

**DOI:** 10.1371/journal.pone.0212215

**Published:** 2019-02-21

**Authors:** Gisel S. Miszczuk, Jesus M. Banales, Andrés E. Zucchetti, Gerardo B. Pisani, Andrea C. Boaglio, Elena Saez, Juan F. Medina, Marcelo G. Roma, Fernando A. Crocenzi

**Affiliations:** 1 Instituto de Fisiología Experimental (IFISE)–Consejo Nacional de Investigaciones Científicas y Técnicas (CONICET), Facultad de Ciencias Bioquímicas y Farmacéuticas–Universidad Nacional de Rosario, Rosario, Argentina; 2 Division of Gene Therapy and Hepatology, CIMA, University of Navarra, Pamplona, Spain; 3 Department of Liver and Gastrointestinal Diseases, Biodonostia Research Institute-Donostia University Hospital, UPV/EHU, CIBERehd, Ikerbasque, Donostia-San Sebastian, Spain; 4 Área Morfología, Facultad de Ciencias Bioquímicas y Farmacéuticas, Universidad Nacional de Rosario, Rosario, Argentina; Texas A&M University, UNITED STATES

## Abstract

In obstructive cholestasis, there is an integral adaptive response aimed to diminish the bile flow and minimize the injury of bile ducts caused by increased intraluminal pressure and harmful levels of bile salts and bilirrubin. Canalicular bicarbonate secretion, driven by the anion exchanger 2 (AE2), is an influential determinant of the canalicular bile salt-independent bile flow. In this work, we ascertained whether AE2 expression and/or activity is reduced in hepatocytes from rats with common bile duct ligation (BDL), as part of the adaptive response to cholestasis. After 4 days of BDL, we found that neither AE2 mRNA expression (measured by quantitative real-time PCR) nor total levels of AE2 protein (assessed by western blot) were modified in freshly isolated hepatocytes. However, BDL led to a decrease in the expression of AE2 protein in plasma membrane fraction as compared with SHAM control. Additionally, AE2 activity (J_OH_-, mmol/L/min), measured in primary cultured hepatocytes from BDL and SHAM rats, was decreased in the BDL group versus the control group (1.9 ± 0.3 *vs*. 3.1 ± 0.2, p<0.005). cAMP-stimulated AE2 activity, however, was not different between SHAM and BDL groups (3.7 ± 0.3 *vs*. 3.5 ± 0.3), suggesting that cAMP stimulated insertion into the canalicular membrane of AE2-containing intracellular vesicles, that had remained abnormally internalized after BDL. In conclusion, our results point to the existence of a novel adaptive mechanism in cholestasis aimed to reduce biliary pressure, in which AE2 internalization in hepatocytes might result in decreased canalicular HCO_3_^-^ output and decreased bile flow.

## Introduction

Bile formation involves an osmotic water flow in response to active solute transport. Bile salts are secreted to the canaliculi through a specific export pump referred to as BSEP (ABCB11) [1], allowing for the generation of the bile salt-dependent fraction of bile flow. The remaining bile salt-independent fraction of bile flow is driven by supplementary solutes secreted at both the canalicular and the ductular levels (the so-called canalicular bile salt-independent bile flow, and ductular bile salt-independent bile flow, respectively) [2].

In addition to bile salt secretion, the hepatocellular canalicular membranes shows active secretion of other organic and inorganic compounds, mainly glutathione [3] and HCO_3_^-^ [4], respectively. Glutathione can be secreted via the organic anion transporter MRP2 (ABCC2) [5], while the efflux of HCO_3_^-^ occurs through a Na^+^-independent Cl^-^/HCO_3_^-^ exchanger (AE2, SLC4A2) [4]. AE2 functions in connection with an apical chloride channel that maintains favorable Cl^-^ gradients and with the canalicular water channel aquaporin 8 (AQP8) [4]. AE2 requires suitable intracellular levels of HCO_3_^-^, which are achieved through both its cotransport with Na^+^ by the electrogenic sodium/bicarbonate (NBCe) symporter in the sinusoidal membrane and its formation, catalized by the intracellular carbonic anhydrase. This latter process is linked to H^+^ extrusion via the Na^+^/H^+^ exchanger (NHE), in both the basolateral (NHE1) and the canalicular (NHE3) membranes, Na^+^ being extruded via the basolateral pump, Na^+^/K^+^-ATPase [4]. ATP dependence of this latter process makes HCO_3_^-^ secretion a concentrative mechanism, thus allowing the generation of osmotic canalicular gradients that drive canalicular water flow [6].

The canalicular secretion of glutathione [3] and HCO_3_^-^ [4] are the main driving force for the generation of the canalicular bile salt-independent bile flow. Canalicular HCO_3_^-^ secretion has been reported to be regulated by glucagon [7]. This pancreatic hormone leads to increased intracellular cAMP levels in hepatocytes, PKA activation, and stimulation of AE2 exchange activity [7], as well as enhanced AQP8-mediated water permeability at the canalicular membrane [8]. Interestingly, there is evidence supporting that, in hepatocytes under basal conditions, the water channel AQP8, the Cl^-^/HCO_3_^-^ exchanger AE2 and the glutathione carrier MRP2 are present in pericanalicular vesicles that migrate to the canalicular membrane upon glucagon stimulation [8].

Obstructive cholestasis represents a wide range of liver pathologies where a mechanical blockage in the bile ducts prevents bile from flowing into duodenum; as a consequence, it leads to retention of biliary constituents in the liver and in the obstructed bile ducts. Among these hepatopathies, gallstone disease secondary to cholelithiasis, biliary atresia, primary sclerosis cholangitis (PSC), and late-stage primary biliary cholangitis/cirrhosis (PBC) represent prototypic pathologies with an obstructive cholestatic component [9].

Common bile duct ligation (BDL) is a useful animal model for the study of changes occurring in obstructive cholestatic pathologies. Most of the changes in the expression of hepatic transport proteins (both in hepatocytes and cholangiocytes) reported in this model are considered as adaptive changes tending to minimize cellular damage induced by retained, potentially toxic, biliary constituents, such as bile salts and bilirubin [10]. These adaptive changes, several of which have been confirmed in patients with obstructive cholestasis due to late-stage PBC [11,12], involve the following: *i*) increased expression of enzymes engaged in the detoxification of biliary constituents, *ii*) inhibition of endogenous bile salt synthesis, *iii*) downregulation of the basolateral uptake systems such as the Na^+^-taurocholate cotransporter protein (NTCP) and the organic anion transporting protein 1 (OATP1), *iv*) downregulation of canalicular transporters BSEP, MRP2 and AQP8, and *v*) increased expression of basolateral export pumps [13]. Interestingly, adaptive changes in hepatocellular transporters were reported to occur early after BDL, in an attempt to prevent irreversible alterations from occurring at earlier times. Indeed, downregulation of the basolateral uptake system NTCP [14], OATP1, OATP3, and OATP4 [15], downregulation of the canalicular export pumps BSEP and MRP2 [16], and upregulation of the basolateral export transporters OSTα/OSTβ [12], MRP3 and MRP4 [15,17], were apparent as soon as 3 days after BDL. Thus far, however, the regulation of AE2 expression and activity in hepatocytes under cholestatic conditions remains to be investigated. Therefore, we evaluated here the expression, localization and activity of AE2 in hepatocytes after BDL in the rat.

## Materials and methods

### Animals and treatments

Male Wistar rats (Harlan) weighing approximately 250 g were housed under a controlled environment (12 hr light/12 hr dark; temperature, 22–24°C), and received a standard diet and water *ad libitum* in the animal facility of the CIMA, University of Navarra. All animal handling and surgical procedures were carried out in strict accordance with the Guide for the Care and Use of Laboratory Animals of the National Institutes of Health and were approved by the Committee of Ethics for Animal Experimentation (CEEA) of the University of Navarra. The surgical procedures were performed under continuous inhalation anesthesia. Isoflurane 5% with oxygen (1 L/min) was used for the induction of anesthesia (2–3 min) in an induction chamber. The rats were then placed in maintenance anesthesia at 1–1.5% isoflurane with 0.6 L/min oxygen. Animals were maintained at a normal body temperature using thermal pads. With rats under inhalation anesthesia, the common bile duct was double ligated close to the hepatic hilum and cut between ligatures. Controls underwent a sham surgery, consisting in exposure, but not ligation, of the common bile duct. BDL was maintained for 4 days, a time period sufficient for adaptive response involving most hepatocellular transporter to occur (see above, Introduction section). After 4 days, a blood sample was taken for biochemical determination of cholestatic and hepatocellular damage markers, and a liver sample was obtained by partial hepatectomy for western blot analysis, histology and immunofluorescence for AE2. Then, the liver was perfused with collagenase type IV (Sigma) for hepatocyte isolation, as previously described [18].

### Biochemical plasma determinations

Plasma levels of total alkaline phosphatase (ALP), bilirubin and transaminases (AST and ALT) were determined in a Hitachi 911 analyzer (Roche/Hitachi, Indianapolis, IN), including a normalizing external control.

### Liver histology

For conventional light microscopy, liver tissues were fixed in 4% neutral buffered formaldehyde solution and embedded in paraffin. Then, 4-μm-thick sections were stained with hematoxylin and eosin. The samples were coded and examined by a pathologist (G.B.P.), who was unaware of the animals’ treatment.

### Measurement of the Na^+^-independent Cl^-^/HCO_3_^-^ anion exchange (AE) activity in primary cultured hepatocytes

The AE2 activity was measured in hepatocytes as the rate of spontaneous recovery of pH_i_, after the induction of intracellular alkalinization, by administration and further withdrawal of the cell-permeant weak acid, propionate. For this purpose, isolated hepatocytes were seeded on sterile 12-mm glass coverslips and cultured in DMEM F-12 + Glutamax (Gibco) culture medium with 10% FBS and penicillin/streptomycin, and cultured for 24 h. After that, the cells attached to the glass coverslips were loaded with the pH-sensitive probe 2´-7´-bis-(2-carboxyethyl)-5(6)-carboxyfluorescein-acetoxymethylester (BCECF-AM), and placed on the thermostatic chamber of a fluorescence microscope, where a mercury lamp excites the BCECF-AM-loaded cells. After an initial perfusion with Krebs-Ringer HCO_3_^-^ buffer (KRB), cells were perfused for 4 min with a KRB-propionate buffer. Fluorescence emission was recorded, and the obtained data were further analyzed using the Aquacosmos software. Rates of pH_i_ recovery was measured as δpH_i_/δt from the tangent to the experimental plot; transmembrane acid fluxes (or equivalent transmembrane base fluxes, i.e. J_OH_-) were calculated as ß_tot_ x δpH_i_/δt, ß_tot_ being the total intracellular buffering power in the presence of CO_2_/HCO_3_^-^, estimated as described [19].

The exchange activity measured as indicated above only assesses the function of AE2 localized in the plasma membrane, which is just a fraction of the total amount of AE2 present in the cell. For AE2 activity to reflect total AE2 content, exocytic targeting of internalized AE2 to the plasma membrane must be promoted by elevating cAMP levels. For this purpose, both KRB and KRB-propionate buffers were supplemented with a “stimulatory mixture” containing the permeant cAMP analogue dibutyryl-cAMP (Sigma-Aldrich, 100 μM), the phosphodiesterase inhibitor, IBMX (Sigma-Aldrich, 100 μM), and the adenylate cyclase activator, forskolin (Sigma-Aldrich, 3 μM). Whereas AE2 activity measured under stimulated conditions reflects total AE2 content, the percentage increase in AE2 activity from basal to stimulated conditions reflects the fraction of AE2 that was internalized under the first condition.

### Analysis of AE2 mRNA expression by quantitative real-time PCR

Freshly isolated hepatocytes were lysed for extraction of total RNA by using the TRI Reagent (Sigma). Extracted RNA was resuspended in diethylpyrocarbonate (DEPC, Sigma)-treated water, and quantified by UV spectrometry using the NanoDrop ND-1000 spectrometer (NanoDrop Technologies). Total RNA (1 μg, denatured at 70ºC during 3 min) was reversed transcribed into complementary DNA (cDNA). Levels of the three AE2 mRNA variants were determined by quantitative real-time PCR on resultant cDNA in an iCycler iQ5 Apparatus (Bio-Rad Laboratories), using specific primers for each variant, i.e. *AE2a*, *AE2b1* and *AE2b2* isoforms. Glyceraldehyde phosphate dehydrogenase (*GAPDH*) was used as the housekeeping gene for normalization of data. AE2 mRNA levels in BDL and SHAM rat hepatocytes were estimated from the cycle threshold (Ct) values obtained for each sample, and normalized to GAPDH mRNA levels according to the following equation: *Relative AE2 mRNA level* = 1.8^(CtGAPDH-CtAe2)^ (an overall PCR efficiency of 80% was assumed).

### Assessment of AE2 subcellular localization

AE2 protein content was evaluated by western blot of total hepatocyte membranes. For this purpose, homogenates from liver samples obtained by 15 up-and-down strokes with a loose-fitting Dounce homogenizer in 4 volumes of 0.3 M sucrose (MP Biomedicals, Solon, OH), containing a mixture of protease inhibitors (Sigma Chemical Co., 1:1000) were subjected to low-speed centrifugation (500g, 10 min) to obtain post-nuclear supernatants. This fraction was then centrifuged at 200,000g for 60 min at 4ºC, yielding the total liver membrane fractions [20].

In order to evaluate AE2 distribution between plasma membrane and microsomal membrane, these two membrane fractions were obtained by differential centrifugation, as previously described [20]. Briefly, the post-nuclear fractions obtained from liver homogenates as described above were subjected to centrifugation at 200,000g for 60 min at 4°C on a discontinuous 1.3 M sucrose gradient. Then, the membrane band was removed, and further purified on a 9% to 60% linear sucrose gradient, to yield the plasma membrane (PM) fraction. The remaining gradient was sonicated and centrifuged at 17,000g for 30 minutes. The resulting supernatant was centrifuged again at 200,000g for 60 min, to yield the intracellular microsomal membrane (IM) fraction.

Proteins in total membrane and PM and IM fractions were determined according to Lowry *et al*. [21] by using bovine serum albumin as standard. Western blots of total membrane and membrane fractions were performed by using a rabbit antibody against mouse AE2 (Alpha Diagnostics), a mouse antibody against ß-actin (Sigma), and the corresponding horseradish peroxidase-conjugated secondary antibodies (Thermo Scientific, Rockford, IL). Protein bands were detected by an enhanced chemiluminescence detection system (Thermo Scientific). Autoradiographs were obtained by exposing the membranes to Amersham Hyperfilm ECL (GE Healthcare Limited, Chalfont St. Giles, UK). Densitometric analysis of the developed bands was performed using ImageJ Software.

To evaluate the intracellular distribution of AE2, a liver lobe was excised 4 days after BDL or SHAM surgery, frozen immediately in liquid nitrogen-precooled isopentane, and stored at −70°C for further immunofluorescent staining and confocal microscopy analysis, as described previously [22]. Liver slices were fixed, permeabilized, and incubated with the antibody against AE2 (1:100, overnight), and canaliculi were delimited by staining occludin (Invitrogen, 1:100, overnight), followed by incubation with the respective Cy2 and Cy3-conjugated secondary antibodies (Jackson ImmunoResearch, 1:200, 2h). Confocal images were obtained by using a Confocal Nikon C1 Plus microscope.

### Statistical analysis

Data belonging to BDL rats were compared to SHAM group using the unpaired Student’s *t*-test, after confirming that the data met appropriate assumptions (normality, homogenous variance and independent sampling). When a non-parametric test was required, the Mann-Whitney *U*-test was employed. A two-tailed *p*<0.05 value was considered statistically significant.

## Results

### Biochemical and histological parameters after 4 days of BDL

**Fig 1:**
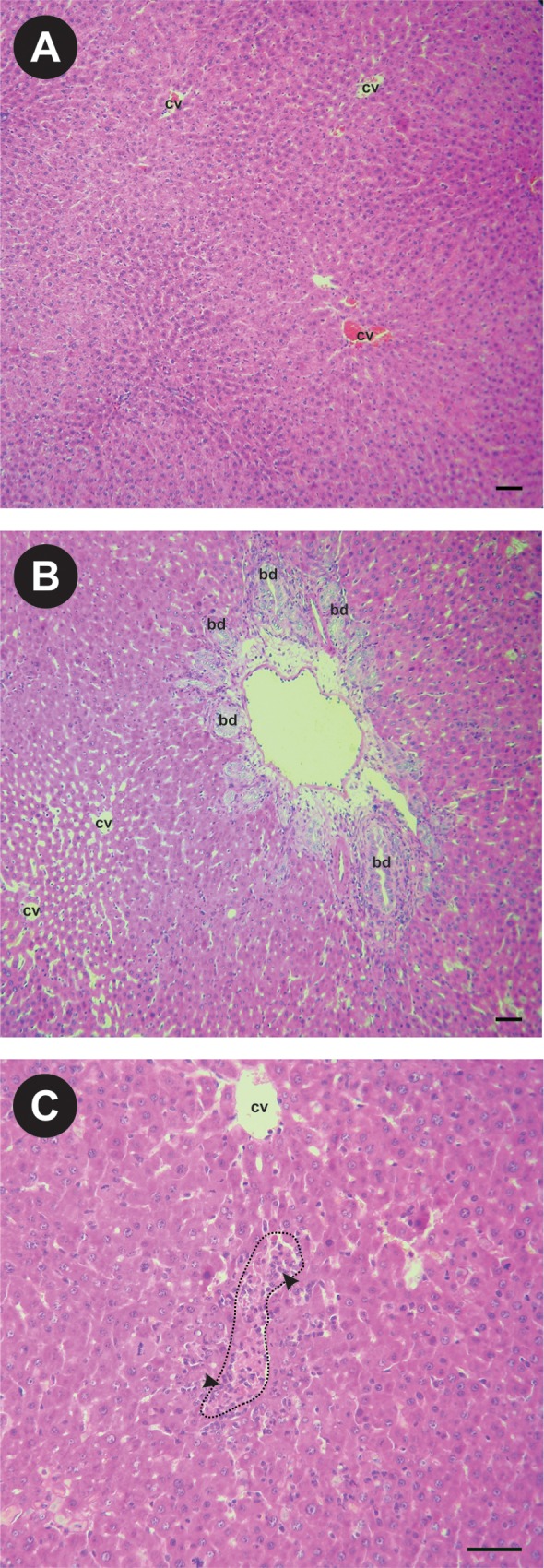
Representative microscopy images of hematoxilin and eosin-stained liver slices from SHAM and BDL animals. (A) Conserved histological architecture corresponding to the SHAM group. (B) Pronounced dilatation and proliferation of bile ducts induced by BDL. (C) Necrotic areas (delineated by a dotted line) infiltrated by neutrofils (arrowheads) in BDL livers. Scale line = 50 μm. cv = central vein; bd = bile duct.

As shown in Table 1, plasma levels of the cholestasis markers ALP and total bilirubin were found elevated after 4 days of BDL. Hepatic transaminase levels (AST and ALT), which are indicative of hepatocyte integrity, were also found to be elevated, in agreement with the focal necrosis observed in hematoxylin and eosin-stained slides (Fig 1C). Effective establishing of the obstructive cholestatic model was also confirmed by the observation of the apparent dilatation and proliferation of bile ducts (Fig 1B).

**Table 1:** Plasma levels of alkaline phosphatase (ALP), total bilirubin, AST and ALT after BDL.

	ALP (U/L)	Bilirubin (mg/dL)	AST (U/L)	ALT (U/L)
**SHAM**	119 ± 15	0.1 ± 0.0	256 ± 31	54 ± 7
**BDL**	602 ± 140*	7.9 ± 1.7*	1413 ± 106*	636 ± 67*

Results are mean *±* SEM

*p<0.05 *vs*. SHAM, *n* = 3 each.

### AE activity in primary cultured hepatocytes

**Fig 2:**
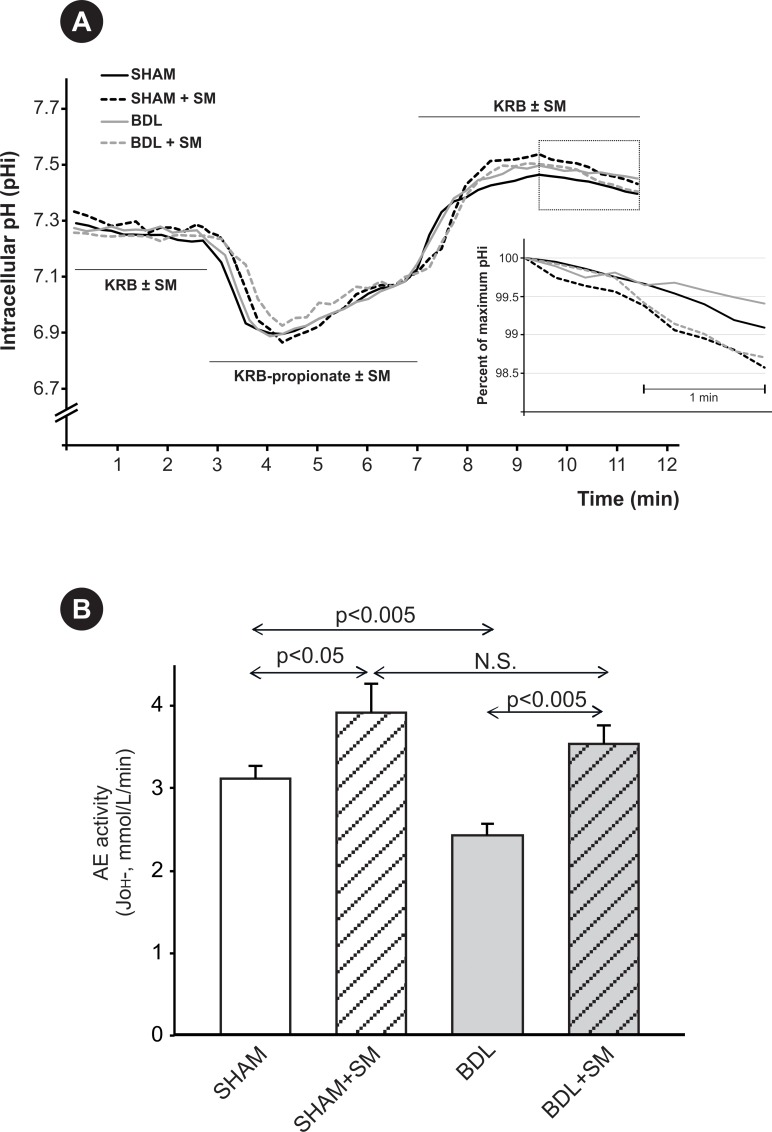
Changes in hepatocyte anion exchange activity induced by BDL. (A) Representative traces of changes in intracellular pH (pHi) of perfused rat hepatocytes showing the recovering acidification that follows the alkalinization elicited by propionate removal, and results from AE2-mediated HCO_3_^-^ secretion (demarcated zone in the curves). Inset graph shows the pH_i_ changes with time, indicative of AE activity, during the phase of recovering acidification, expressed as the percent of the initial, maximum pH_i_ in BDL and SHAM hepatocytes, under the presence or absence of a stimulatory mixture (SM: dibutyryl cyclic AMP, IBMX and forskolin) in the perfusion buffers. (B) Anion exchange (AE) activity, expressed as J_OH_-, in primary cultured hepatocytes from BDL and SHAM rats, in the presence or absence of SM in the perfusion buffers. Results are mean *±* SEM, *n* = 20 to 60 cells per preparation, from 3 independent cellular preparations per experimental group.

Hepatocytes isolated from BDL and SHAM rats were cultured for 24 h to allow for a correct cell attachment and reproducible AE activity measurements, as previously reported in hepatocytes [23] and hepatic cell lines [24]. Fig 2A shows representative traces of changes in pHi of perfused SHAM and BDL rat hepatocytes showing the recovering acidification that follows the alkalinization elicited by propionate removal (see Materials and methods), and results from AE2-mediated HCO_3_^-^ secretion (demarcated zone in the curves). Inset graph shows the pH_i_ changes with time during the phase of recovering acidification, expressed as the percent of the initial, maximum pH_i_ in hepatocytes; the slope of the curves, indicative of the rate of pHi recovery, directly depends on AE activity. This parameter was decreased in the BDL group (Fig 2A, inset). However, under cAMP-stimulating conditions, the rate of pHi recovery was increased to the same extent in both SHAM and BDL hepatocytes. Indeed, calculated J_OH_- values (Fig 2B) indicated that BDL leads to a significant decrease in baseline AE activity. Under cAMP-stimulating conditions, however, activities of both SHAM and BDL hepatocytes were increased above baseline values, reaching similar values in both BDL and SHAM hepatocytes. These findings strongly suggest that the decrease in AE activity after BDL might be associated to abnormal internalization of AE2; after stimulation with cAMP, AE2-containing intracellular vesicles would be reinserted into the canalicular membrane, allowing for restoration of the AE activity.

### AE2 mRNA expression levels

The expression of all the hepatic isoforms of *AE2* mRNA (i.e. *AE2a*, *AE2b1* and *AE2b2* variants), was quantified by quantitative Real-Time PCR in isolated hepatocyte samples. *AE2* mRNA levels in the BDL group were not significantly different from those in SHAM group (Fig 2A), indicating that there is no change in *AE2* expression after BDL.

### Intracellular distribution of AE2

**Fig 3:**
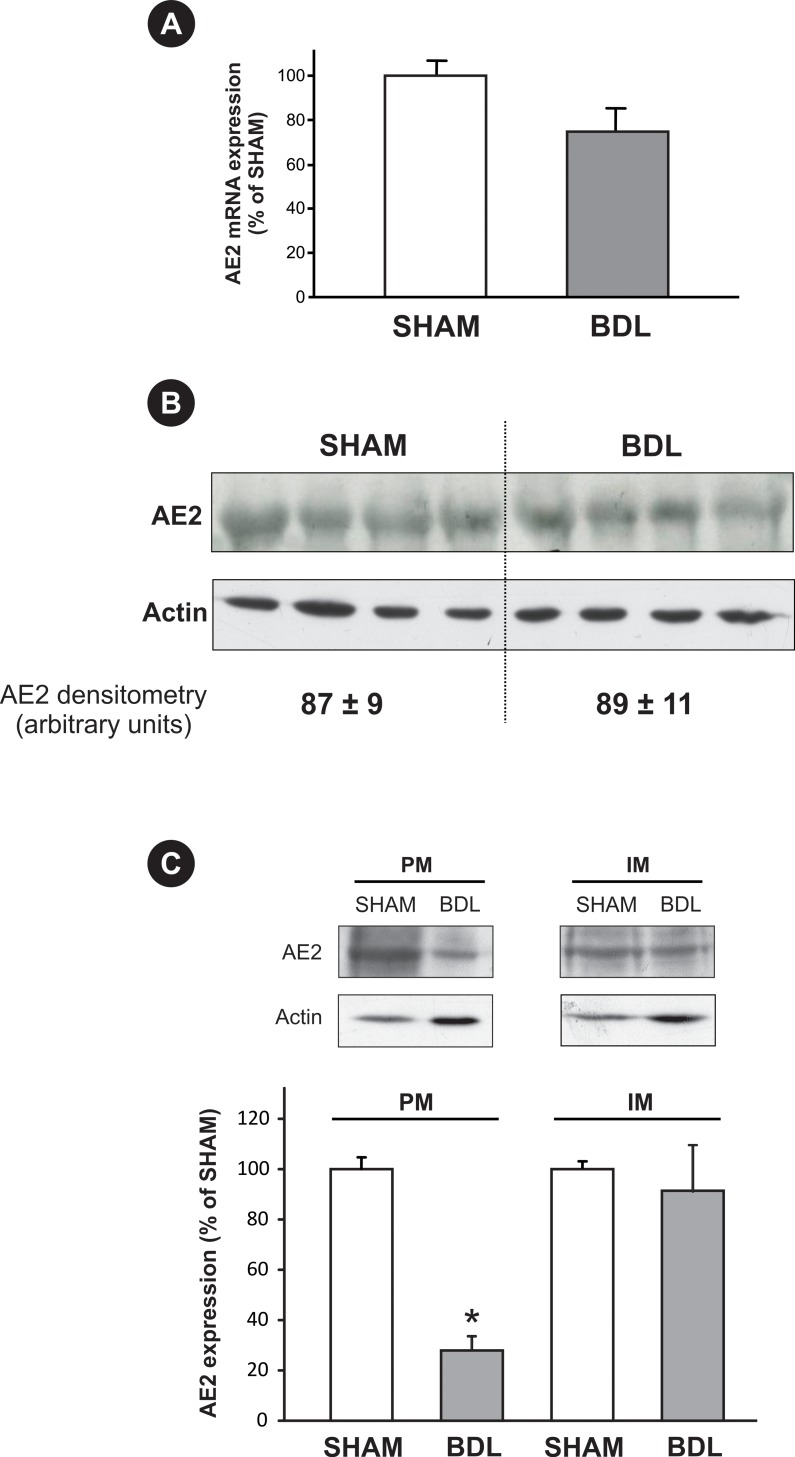
Changes in hepatocellular AE2 expression induced by BDL. (A) Expression of *AE2* mRNA variants in hepatocytes isolated from BDL and SHAM rats (values are given as percentage of those in SHAM control). Results are mean *±* SEM, *n* = 4 each. (B) Expression of AE2 protein in total membrane obtained from liver homogenates. Differences in sample loading were corrected by the densitometric signal of the respective β-actin band. An arbitrary value of 100 was assigned to the SHAM band with the highest densitometric signal. Results are mean *±* SEM, *n* = 4 each. (C) Expression of AE2 protein in plasma membrane (PM) and intracellular microsomal membrane (IM) fractions. Differences in sample loading were corrected by the densitometric signal of the respective β-actin band. Densitometric data were expressed as percentage of SHAM values. Results are mean *±* SEM, *n* = 6 each. *p<0.05 *vs*. SHAM.

**Fig 4:**
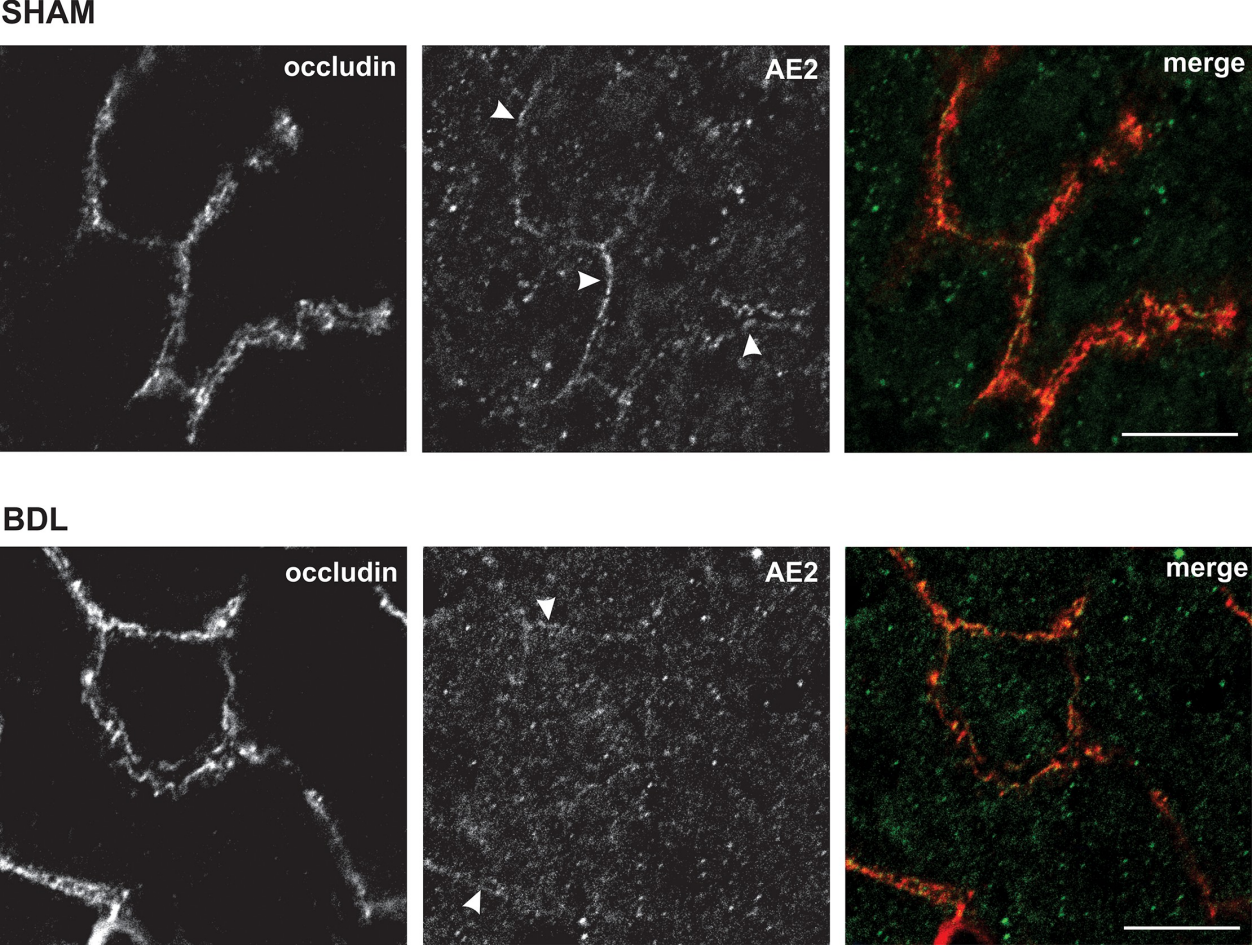
Changes in subcellular localization of AE2 induced by BDL. Representative confocal images of SHAM (upper panels) and BDL (lower panels) liver sections. Fluorescent immunostaining was performed for AE2 (green) and occludin (red). In SHAM, AE2 is localized in the canalicular zone (arrowheads, demarcated by occludin) and also in some intracellular vesicles (upper panels). BDL induced a clear internalization of AE2, thus leading to a significant diminution of AE2 in the canalicular zone (arrowheads, lower panels) and the consequent increase in the number of AE2-positive intracellular vesicles.

Fig 3C shows that AE2 protein is encountered in both IM and PM fractions. BDL leads to a decrease in the expression of AE2 protein in PM, with no modification of its expression in IM. This decrease in the expression of AE2 in PM, together with the lack of decrease in the expression of AE2 in total membrane (Fig 3B), is consistent with deficient apical insertion of AE2-containing vesicles (and/or with endocytic AE2 internalization) following BDL. The lack of any detectable increase in AE2 expression in IM after BDL appears to be related to the fact that, in hepatocytes under basal conditions, AE2 mainly resides in subapical vesicles; this would induce changes in the expression of AE2 in IM fraction beyond the sensitivity of the Western blot assay. A main subapical AE2 localization in BDL group was also apparent by confocal microscopy, where the transporter is barely visible in the canalicular membrane of some canaliculi, but mainly present in numerous intracellular vesicular structures (Fig 4, arrows, middle bottom panel). On the other hand, in the SHAM group (Fig 4), AE2 staining is clearly visible in the canalicular membrane, as well as in some intracellular vesicles (Fig 4, arrows, middle upper panel).

## Discussion

Our results provide strong evidence for obstructive cholestasis leading to decreased anion exchange activity in hepatocytes, due to low canalicular expresion of AE2, which is seemingly redistributed to pericanalicular intracellular vesicles. While neither *AE2* mRNA levels nor AE2 protein expression in total membranes were modified in hepatocytes after 4 days of BDL, the expression of AE2 was found to be decreased in the PM fraction. Our finding of recovery of the AE activity in BDL hepatocytes to SHAM values upon cAMP stimulation is consistent with cAMP-induced exocytic insertion of pericanalicular AE2-containing vesicles. Actually, AE2 mainly resides in subapical vesicles [25], that can be exocytosed to the canalicular membrane on demand, as reported to occur under stimulation with glucagon, a cAMP-elevating agent [26]. It was also reported that, as soon as after 2 days of BDL, glucagon-stimulated cAMP synthesis is impaired by bile salt-dependent PKC-α activation [27,28], at least in part by a decrease in glucagon receptors in the sinusoidal membrane [29]. It was also shown that pro-inflammatory cytokines, which are elevated both in blood and in hepatic tissue after BDL [30], diminished cholangiocyte cAMP synthesis and AE2 membrane insertion, via generation of NO [31]. Interestingly, also in hepatocytes, BDL diminishes cAMP production [27] and increases the levels of NO induced by cytokines [32]. Therefore, alteration of the normal targeting of this transporter by impaired cAMP exocytic stimulation could contribute to the decrease in the PM expression of AE2 and diminished AE activity observed in our experiments.

In addition to altered targeting to its membrane domain, AE2 might undergo exacerbated endocytic internalization. AE2 is localized in cholesterol- and caveolin-enriched "raft" microdomains in the canalicular membrane [35]. It was recently described that, in estradiol 17ß-d-glucuronide-induced cholestasis, the canalicular transporters BSEP and MRP2 undergo a shift from raft to non-raft canalicular microdomains, from where they can be endocytosed in a clathrin-dependent fashion [33]. Since PKC-α activation was reported to be crucial in estradiol 17ß-d-glucuronide-induced transporter endocytosis, this mechanism is also likely to occur in BDL rats. In line with this, an increased activation of this kinase by translocation from the cytosol to the membrane fraction was reported in hepatocytes isolated after only 2 days of BDL [27], a phenomenon attributed to the intracellular accumulation of bile salts due to the obstructive process [34].

Irrespective of the mechanisms involved, an adaptive downregulation of AE2 function in obstructive cholestasis may be functionally associated with simultaneous downregulation of AQP8 [20], an apical water channel that shares with AE2 the same intracellular vesicles [25,35] and the same pro-exocytic stimulus via the glucagon/cAMP/PKA signaling pathway [8,36,37]. However, the fate of these transporters during cholestasis seems to be different; whereas AQP8 protein expression drops via post-transcriptional mechanisms by 3 days of the obstructive process [20], transcriptional expression and total levels of AE2 protein remained unaltered even after 4 days of cholestasis. This difference may be explained by different mechanisms or timings of intracellular degradation; while AE2 degradation mechanisms have not been characterized yet, AQP8 proteolysis is lysosomal in nature under cholestatic conditions [38]. Differences in the breakdown susceptibility has also been reported for MRP2 and BSEP in obstructive cholestasis despite both transporters share the same pericanalicular vesicles, with BSEP being more resilient to intracellular degradation [39]. Regardless the causes involved, a differential degradation status may explain why AE2 is responsive to cAMP in hepatocytes from animals with extrahepatic cholestasis (see Fig 1), but AQP8 is not [20]. Trafficking to lysosomes is regarded as irreversible [40], so that transporters entering this pathway can no longer be recycled back to the apical membrane. A similar irreversible phenomenon occurs with MRP2 in endotoxin-induced cholestasis; MRP2 can only be recruited for apical reinsertion shortly after the cholestatic insult, when MRP2 remains associated with early, but not with late endosomal compartments [41].

Although it is difficult to establish with certainty how important is the downregulation in AE2 activity for the adaptive reduction of bile production under biliary obstructive conditions, an analysis of the potential contribution of HCO_3_^-^ output to bile flow generation may provide some hints. It has been reported that, following bile flow obstruction, a decrease in bile flow of 80%-90% occurs [42]. Since HCO_3_^-^ was proposed to play a similar quantitative role in the generation of the canalicular bile salt-independent bile flow to glutathione [6], and the biliary secretion of both osmotic agents accounts for at least 50% of bile flow in the rat [43], the downregulation of AE2 activity reported here should be indeed relevant to the adaptive response aimed to decrease canalicular bile production in obstructive cholestasis. This is more so considering that most of the bile generation fall is expected to occur at expense of the bile salt-independent fraction of bile flow, since bile salt output is better preserved in cholestasis [44]. The presumed reduction in canalicular bile flow generation caused by this change would act in concert with other similar changes in canalicular transporters involved in bile formation (*e*.*g*., MRP2, BSEP). This should help to protect hepatocellular tight junctional integrity from the increase in biliary pressure, and to prevent biliary infarcts leading to leakage of bile containing millimolar concentrations of bile acids into the parenchyma via disrupted canals of Hering, thus triggering bile salt-induced hepatocyte cell death [45]. These adaptive modulations in response to obstructive cholestasis may explain why it is needed several weeks or months of biliary obstruction for irreversible liver damage to occur [46].

In conclusion, our results indicate a downregulation of AE2 exchange activity due to AE2 internalization, as a novel adaptive mechanism in cholestasis. Our results may contribute to a deep understanding of the mechanisms involved in this adaptive response, and this appears relevant to envisage new therapeutics options aimed to enhance this beneficial response, and preserve liver integrity in chronic cholestasis.

## Supporting information

S1 Fig Raw, uncropped images of the western blots reported in Fig 3. B and C refer to western blot shown in Fig 3B and 3C, respectively. Lines indicated as S and B correspond to SHAM and BDL groups, respectively. PM = plasma membrane. IM = intracellular membrane.
(TIF)
