# Supplementary figures and images for: Adaptive downregulation of Cl-/HCO3- exchange activity in rat hepatocytes under experimental obstructive cholestasis
Source: PLoS One. 2019 Feb 21;14(2):e0212215. doi: 10.1371/journal.pone.0212215 (PMC6383990; doi:10.1371/journal.pone.0212215)

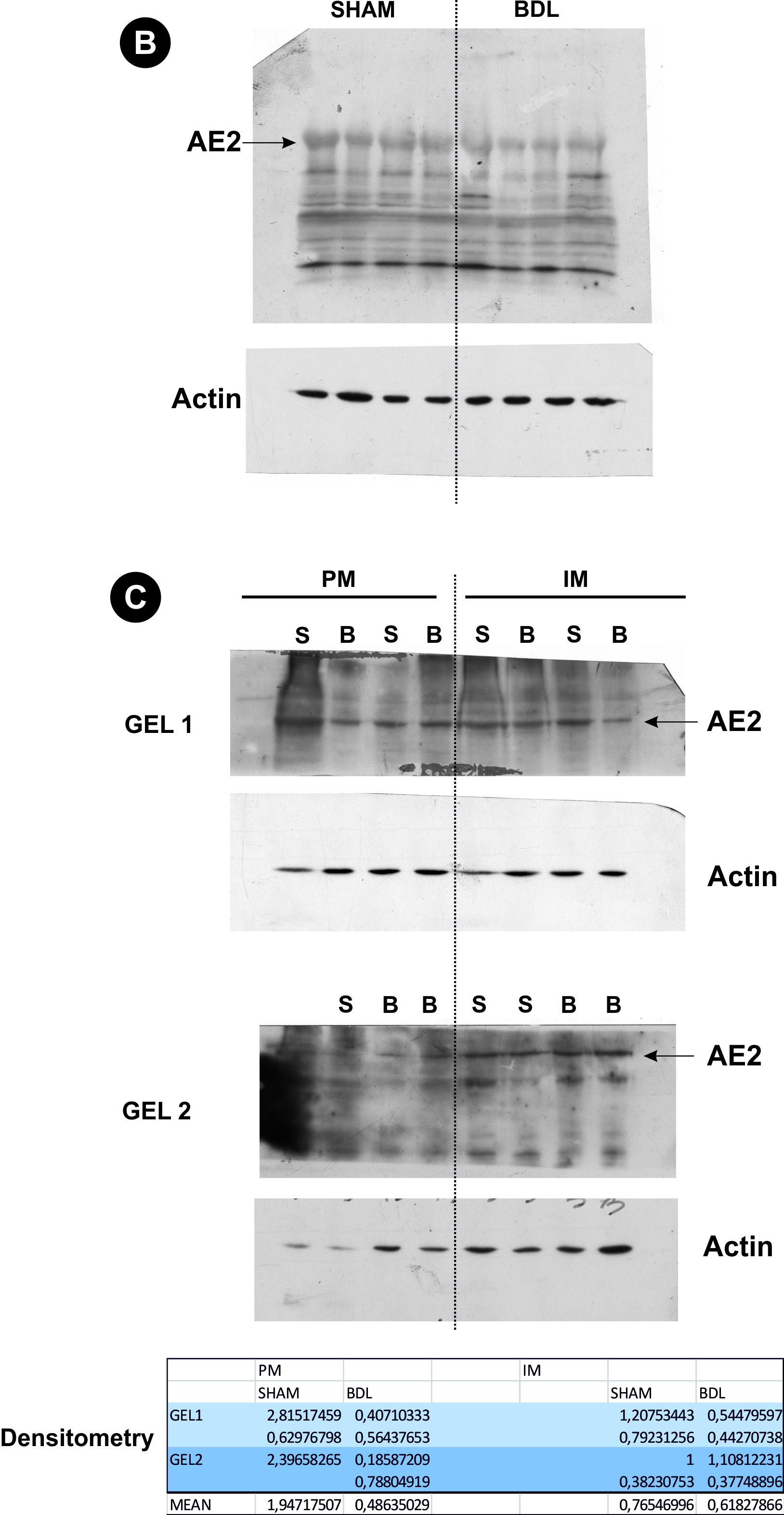

Supplement: S1 Fig — B and C refer to western blot shown in Fig 3B and 3C, respectively. Lines indicated as S and B correspond to SHAM and BDL groups, respectively. PM = plasma membrane. IM = intracellular membrane. (TIF) [file pone.0212215.s001.tif]
